# Sow Contact Is a Major Driver in the Development of the Nasal Microbiota of Piglets

**DOI:** 10.3390/pathogens10060697

**Published:** 2021-06-03

**Authors:** Pau Obregon-Gutierrez, Virginia Aragon, Florencia Correa-Fiz

**Affiliations:** 1 IRTA, Centre de Recerca en Sanitat Animal (CReSA, IRTA-UAB), Campus de la Universitat Autònoma de Barcelona, Bellaterra, 08193 Barcelona, Spain; pau.obregon@irta.cat (P.O.-G.); virginia.aragon@irta.cat (V.A.); 2 OIE Collaborating Centre for the Research and Control of Emerging and Re-emerging Swine Diseases in Europe (IRTA-CReSA), Bellaterra, 08193 Barcelona, Spain

**Keywords:** piglets’ nasal microbiota, artificial rearing, raising environment, sows, bacterial diversity

## Abstract

The nasal microbiota composition is associated with the health status of piglets. Sow-contact in early life is one of the factors influencing the microbial composition in piglets; however, its impact has never been assessed in the nasal microbiota of piglets reared in controlled environmental conditions. Nasal microbiota of weaning piglets in high-biosecurity facilities with different time of contact with their sows (no contact after farrowing, contact limited to few hours or normal contact until weaning at three weeks) was unveiled by 16S rRNA gene sequencing. Contact with sows demonstrated to be a major factor affecting the nasal microbial composition of the piglets. The nasal microbiota of piglets that had contact with sows until weaning, but were reared in high biosecurity facilities, was richer and more similar to the previously described healthy nasal microbiota from conventional farm piglets. On the other hand, the nasal communities inhabiting piglets with no or limited contact with sows was different and dominated by bacteria not commonly abundant in this body site. Furthermore, the length of sow–piglet contact was also an important variable. In addition, the piglets raised in BSL3 conditions showed an increased richness of low-abundant species in the nasal microbiota. Artificially rearing in high biosecurity facilities without the contact of sows as a source of nasal colonizers had dramatic impacts on the nasal microbiota of weaning piglets and may introduce significant bias into animal research under these conditions.

## 1. Introduction

The dynamic ensemble of microbiological communities inhabiting animal body sites is called microbiota [1], and comprises many different types of microorganisms that provide the host with an important set of functions [2,3,4]. Studies unraveling the many positive aspects of the microbiota on different animal hosts (such as metabolic benefits, immune system maturation, and protection against pathogens) [1,5,6,7], together with the fact that the microbiota comprises potential pathogens [1], have put this area in the focus of microbiological research during recent years. Many studies have shown that a stable, specific and diverse community of commensal microbes is associated with health in humans [8], but also in different animals [2,6,7]. On the other hand, alterations on beneficial bacteria can result in overgrowth of opportunistic pathogenic bacteria and predispose to disease [1]. However, most of these studies have been carried out on humans, leaving animal microbiota less assessed [8]. Animal microbiota composition varies among individuals due to the action of many factors, such as the environment, host-genetics, the diet, the use of antimicrobials and the maternal transmission in early life, among others [1,6,7,9].

Among animal farming, pig industry is one of the most important worldwide, since pork is one of the world’s most consumed meats [10]. Antimicrobials have been widely used for many years in swine production with a great success controlling bacterial diseases, but antimicrobial resistance has generated a strong institutional call for the reduction of antimicrobial use in farm animals [11,12]. In addition, antimicrobials have the potential to disrupt the beneficial microbial communities of the microbiota [7,13,14]. Therefore, alternative tools to control diseases while ensuring balanced costs and low economic loses are of great interest [15], including those directed to the development and maintenance of healthy microbial communities [2,7].

The largest animal microbial ecosystem is the gastrointestinal tract, which has been the focus of most microbiota studies, because of its impact on the host [4,7,14,16,17]. Nevertheless, other microbial niches (nasal, oral, skin, vagina, etc.) may also play important roles [17,18], and need more consideration. The upper respiratory tract, in particular the nasal cavity, is the entry way of many respiratory pathogens. Therefore, understanding the nasal microbiota and the factors that modulate its composition can be essential for improving the control of respiratory infections [15]. Furthermore, early life conditions can determine the development and subsequent composition of the microbiota and affect the health status later in life [18].

Recently, some studies have correlated the pig microbiota composition with health or disease [7,14,18,19]. This is especially important at weaning, which is a critical moment due to the changes in feeding, antimicrobial treatments, and physical and social environment [6,7,14,15,19]. The microbial composition at this moment is still unstable and can play a key role in the subsequent health status of the animals [5,7,18,19,20,21,22]. Later, the microbiota constitutes a stable and resilient community that is able to resist some perturbations [15]. Hence, during the first period of life, when the microbiota can undergo many fluctuations, it may be important to favor its stabilization with beneficial communities in order to wean healthier and more resilient piglets.

Several studies have established that correlations between the microbiota of sows and their piglets depend on different aspects such as the delivery mode, feeding and direct contact between the litter and sow [6,7,14,23,24,25]. Initial colonization of piglets is mainly derived from vaginal microbiota in natural delivery, and later from skin, food or the environment [5,7,15,24,26]. However, the nasal microbiota has not been the focus of these studies. On the other hand, some disease models for endemic pathogens require piglets with no contact with the sows and are therefore artificially reared; the so-called snatch-farrowed colostrum-deprived piglets or the caesarean-derived colostrum-deprived models [27,28,29]. Isolation facilities and high biosafety level facilities, which ensure a particularly clean environment, are sometimes used to house these highly susceptible pig models. It is clear that depriving the newborn piglets from colostrum, their initial source of immunity, has a big impact on the piglet health [7]. Nevertheless, the impact of this artificial environment and rearing conditions on the microbiota of the piglets has never been evaluated, especially focusing on nasal microbiota. Therefore, the aim of this study was to assess how a reduced contact with sows and the high biosecurity conditions where piglets are raised affect the nasal microbiota composition of piglets at weaning.

## 2. Results

### 2.1. Sample Collection and Sequencing

**Figure 1:**
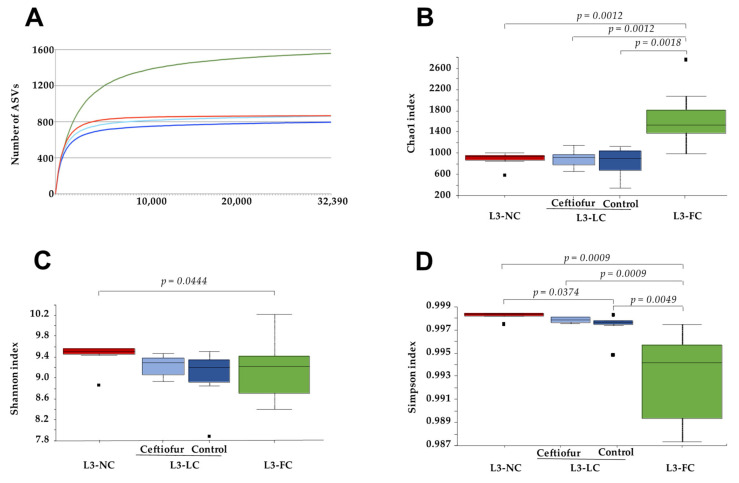
Alpha diversity of the nasal microbiota of 3–4-week-old piglets raised under BSL3 conditions with different degree of contact with their sows. Alpha diversity was measured at a depth of 32,392 by observed ASVs (**A**), Chao1 (**B**), Shannon–Weaver (**C**) and Simpson (**D**) indices for the different groups under study: piglets with no contact with the sow except for the birth canal (L3-NC, in red); piglets with limited contact of less than 12 h (L3-LC, in blue) and those that had full normal contact with their sows (L3-FC, in green). Group L3-LC included animals treated with ceftiofur (‘Ceftiofur’, in light blue) or non-treated (‘Control’, in dark blue). Significant *p* values from Kruskal–Wallis pairwise tests are depicted.

Nasal samples were taken at three to four weeks of age (common age of weaning) from piglets raised either in Biosafety Level 3 (BSL3) facilities or in conventional farms, as depicted in Table 1. To examine the effect of the sow in the nasal microbiota of the offspring, piglets were allowed to have different degree of contact with their sows. Thus, piglets grown in BSL3 conditions (L3) belonged to three different groups: one group of snatch-farrowed colostrum-deprived piglets, taken at delivery in farms with no contact with the dams except for the birth canal (no-contact, L3-NC); a second group with less than 12 h of contact with the sows after birth in the farm, before they were transferred to the BSL3 facilities for housing (limited-contact, L3-LC); and a third group of piglets that were born and raised in the BSL3 facilities and stayed with their sows until sampling (full-contact, L3-FC). In addition, piglets in group L3-LC belonged to two subgroups depending on the treatment at birth with crystalline ceftiofur (treated or control). Total DNA was extracted from nasal swabs and the V3-V4 region of the 16S gene was amplified and sequenced. The sequences from the nasal swabs of a group of conventional piglets at weaning, born and raised at the farm (farm born-farm raised, FB-FR) from three farms with confirmed good health status (no clinical signs in maternity and nursery units) from Catalonia (GM, PT and VL) from a previous study [18], were also included in the analysis. A total of 31,245,358 paired-end reads were obtained from five different runs. Paired-end reads were joined, quality filtered and sorted into amplicon sequence variants (ASVs). An amount of 29,794 ASVs were obtained, with a total frequency of 3,569,216 sequences (mean frequency of 71,384.32 per sample). The rarefaction curves were built (Figure 1A and Supplementary Figure S1A) to determine the optimal depth where the plateau was reached and, hence, the microbial communities were well represented. In order to normalize data (rarefaction), one sample from L3-LC group was removed due to its low coverage (5,320 sequences in comparison with 73,555 for the rest of the samples) to mitigate possible biases related to uneven sampling depths [30].

### 2.2. Alpha Diversity of the Nasal Microbiota of Piglets with Different Degree of Sow Contact

To assess the effect that sows have on the nasal microbiota of their piglets, samples from piglets raised under the same BSL3 conditions but with different sow–contact time were compared. Samples were rarefied to an even depth of 32,392 sequences/sample and alpha diversity was evaluated through different metrics. The mean number of ASVs at this depth was 860 for L3-NC, 803 for L3-LC and 1470 for the L3-FC group (Figure 1A). The highest richness of L3-FC group was confirmed when estimated through Chao1 index (Kruskal–Wallis test, 999 permutations, *p* < 0.05, Figure 1B). However, when evenness was taken into consideration, the group showing significantly higher diversity was L3-NC, either by Shannon (Figure 1C) or Simpson (Figure 1D) indices. The alpha diversity variability within each group increased with the length of sow–piglet contact, especially when including evenness (Figure 1C,D).

The comparison of ceftiofur-treated and non-treated piglets within the L3-LC group did not show significant differences in alpha diversity (*p* = 0.87, observed features; *p* = 0.75, Chao1; *p* = 0.63, Shannon; *p* = 0.42, Simpson).

### 2.3. Nasal Microbiota Composition of Piglets Differed Depending on the Degree of Contact with Sows

**Figure 2:**
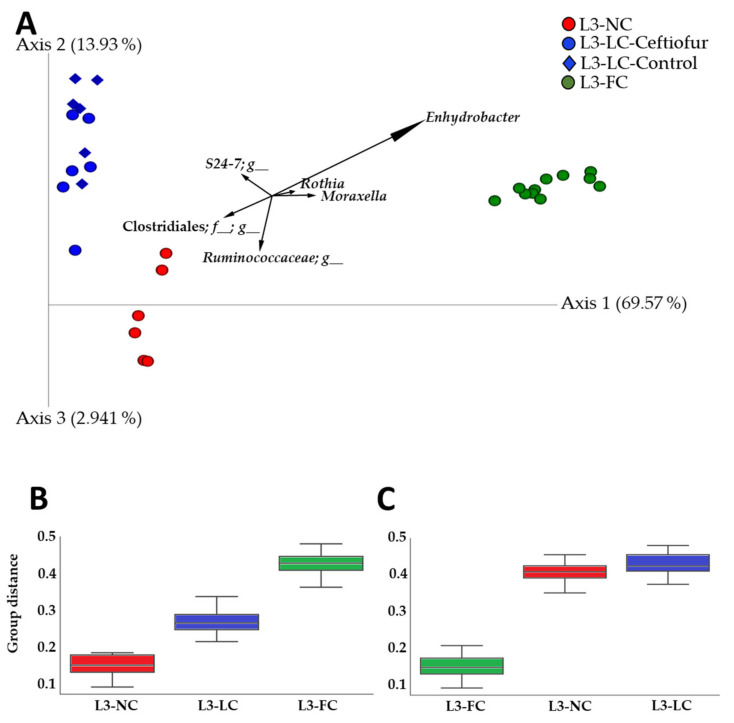
Beta diversity of the nasal microbiota of 3–4-week-old piglets raised under BSL3 conditions with different degree of contact with their sows. (**A**) Principal coordinate analysis representing beta diversity of the nasal microbiota of piglets raised in BSL3 conditions with no contact with sows except the birth canal (L3-NC, in red), limited contact of less than 12 h (L3-LC, in blue) and full normal contact (L3-FC, in green) was computed through weighted UniFrac analysis. The six most relevant genera explaining the differences among groups are plotted in the PCoA space. The length of each of the taxonomic vectors approximates the variance of each taxon throughout the samples. Group clustering distance to L3-NC (**B**) and to L3-FC (**C**) computed by PERMANOVA pairwise test with weighted UniFrac distance matrix (*p* = 0.001 in all cases).

To unravel compositional differences between groups, beta diversity was estimated with unweighted and weighted UniFrac phylogenetic distances. Principal coordinate analysis (PCoA) was performed to assess the degree of clustering among groups and the percentage of variation explained by each clustering was estimated (Adonis function from the Vegan package). Sample clustering of the three groups under study was statistically significant when evaluated with unweighted and weighted UniFrac distance matrices (PERMANOVA, *p* = 0.001 in all cases). Sow–piglet contact explained 43% and 82% of the clustering in the unweighted and weighted analysis, respectively (Adonis, *p* = 0.001). A PCoA biplot was built to represent the most relevant genera that describe the separation in the same PCoA space together with the samples (Figure 2A). In the biplot analysis, *Enhydrobacter*, *Moraxella* and *Rothia* vectors were directed to the PCoA space occupied by samples from L3-FC group, indicating that these genera might be associated to this group. The vectors of two genera from *Firmicutes* (from *Clostridiales* order and *Ruminococcaceae* family) were pointing to the L3-NC space and a genus from *Bacteroidetes* (from family *S24-7*, also known as *Muribaculaceae*) to the L3-LC space. Interestingly, L3-NC and L3-LC groups were qualitative and quantitatively more similar between them than to L3-FC, as indicated by the lower distances between them (Figure 2B,C).

Ceftiofur treatment within L3-LC did not influence the nasal microbiota composition under these conditions (Figure 2A). This was confirmed by comparing only these two subgroups, ceftiofur treated and non-treated, from L3-LC under beta diversity metrics (PERMANOVA, *p* = 0.445 and *p* = 0.146 for unweighted and weighted UniFrac, respectively).

The effect of contact between sow and piglets was also evaluated by comparing the clustering between the group of ‘positive contact’ (L3-LC and L3-FC together) and ‘negative contact’ (L3-NC). Moreover, we calculated the effect of ‘normal sow–contact’ (L3-FC) versus ‘altered contact’ (L3-NC and L3-LC together). The percentage of explanation of these clusters were lower in positive or negative contact (12% and 18% for unweighted and weighted Unifrac, *p* < 0.01) than for normal vs. altered contact (31% and 68%, *p* = 0.001), indicating that the time of contact with the sows is a stronger driver of microbiota composition than the mere existence of this contact.

### 2.4. Comparison of the Nasal Microbiota Core from Animals with Variable Sow Contact

**Figure 3:**
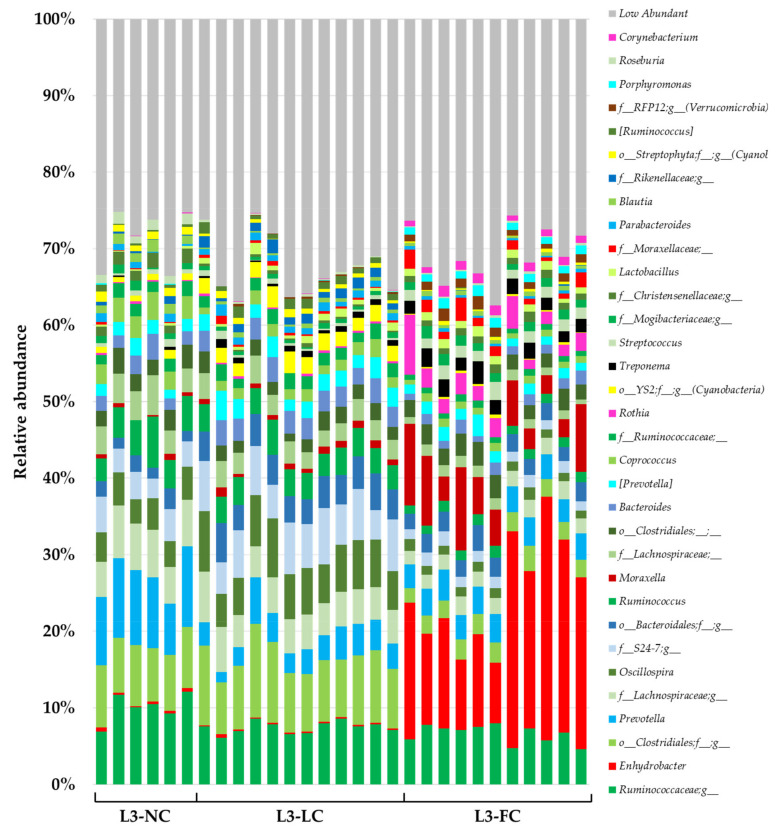
Taxonomical composition at genus level of the nasal microbiota from piglets raised in BSL3 conditions with variable contact with sows. Only taxa with global relative abundance higher than 1% in at least one group is color-coded. All genera with less relative abundance than 1% have been grouped and are shown in grey. L3-NC, no contact with the sows except the birth canal; L3-LC, limited contact of less than 12 h; and L3-FC, full normal contact until weaning at 3 weeks of age. Genera belonging to most relatively abundant phyla have been colored in common color-scheme to simplify its visualization; green for *Firmicutes*, blue for *Bacteroidetes*, and red for *Proteobacteria*. The legend shows the genera ordered by global relative abundance from bottom to top.

To unravel the composition of the different groups with variable sow contact, the ASVs were taxonomically classified. Three phyla comprised 88% of the global relative abundance: *Firmicutes* (49%), *Bacteroidetes* (23%) and *Proteobacteria* (16%). Within the *Firmicutes*, and *Clostridiales* orders it was the most relatively abundant (41% of the global relative abundance), comprising of mainly two families: *Ruminococcaceae* and *Lachnospiraceae*. Among the classified genera, the most relatively abundant were *Oscillospira*, *Ruminococcus* and *Coprococcus*. *Firmicutes* were similarly represented by *Bacillales* and *Lactobacillales* orders, such as *Streptococcus*, *Lactobacillus* and *Staphylococcus*. The most relatively abundant families among *Bacteroidetes* phylum belonged to *Bacteroidales* order (22% of the global relative abundance), where *Prevotellaceae* (mainly represented by *Prevotella* genus) and *S24-7* were the most relatively abundant. Most *Proteobacteria* were classified as *Gammaproteobacteria*, where *Moraxellaceae* was the most relatively abundant family within this phylum and the second globally, mainly composed of *Enhydrobacter* and *Moraxella* genera. Regarding other phyla, some relatively abundant families were *Micrococcaceae* (*Actinobacteria*), mainly represented by the genus *Rothia*; and *Spirochaetaceae* (*Spirochaetes*), mainly represented by *Treponema*. Taxonomical composition at genus level of the most relatively abundant taxa globally (>1% relative abundance in one group at least) is depicted in Figure 3. The full list of the taxonomical assignment of the most relatively abundant taxa from the different groups is included in Table S1, and the corresponding plots at phyla and family levels can be found in Supplementary Figure S1.

**Figure 4:**
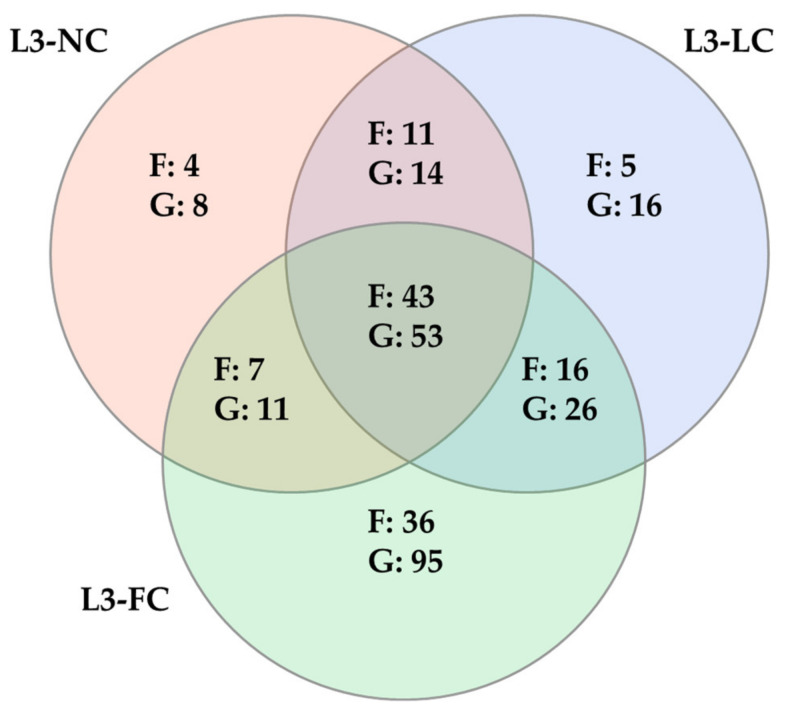
Venn diagram of the shared and specific taxa from the nasal microbiota of piglets raised under BSL3 conditions with different degree of contact with their sows: no contact except the birth canal (L3-NC, in red); limited contact of less than 12 h (L3-LC, in blue) and full normal contact until weaning at 3 weeks of age (L3-FC, in green). Taxa was analyzed at family (F) and genus (G) levels. Only taxa present in more than 80% of samples in a group were included.

The core microbiota was defined as the taxa represented in more than 80% of samples in each group at two different levels: family and genus. The number of core taxa found was 65, 75 and 102 families and 86, 109 and 185 genera for L3-NC, L3-LC and L3-FC, respectively. The number of shared and exclusive taxa among groups is represented in Figure 4, while the full list of taxa is available in Table S2. A common core for the three groups was composed of 43 families and 53 genera, which included the majority of most relatively abundant families and genera (26 out of 33 of families, and 26 out of 34 genera), previously defined as those taxa present in over 1% relative abundance in at least one group (included in Table S1). The two groups with sow-contact, L3-LC and L3-FC, had more taxa in common than with L3-NC. Finally, L3-FC held the highest number of exclusive taxa.

In order to find differentially abundant taxa in the different groups with different sow contact, analysis of composition of microbiomes (ANCOM) and a discrete false-discovery-rate (DS-FDR) analysis were performed. The taxa with more than 1% of relative abundance in at least one group and found differentially abundant in either test, are shown in Table 2. Among the highest relative abundance taxa, *Firmicutes* was detected in higher abundance in altered sow–piglet contact groups, attributed mainly to *Lachnospiraceae* and *Ruminococcaceae* families (*Clostridiales* order, with some exceptions). *Bacteroidales* order (*Bacteroidetes*) was also increased in these groups, with higher abundance of family *S24-7*, or Prevotella in L3-NC and p-2534-18B5 in L3-LC. Contrary, the order *Flavobacteriales* or the genus *Porphyromonas* were more represented in L3-FC. On other hand, an increase in the relative abundance of *Actinobacteria* and *Proteobacteria* (mainly *Moraxella* and *Enhydrobacter*) was observed in L3-FC group, most of these observations validated with both approaches. Similarly, *Bacillales* and *Lactobacillales* orders (*Firmicutes*) were increased when the time of sow–piglet contact was longer (detected only with DS-FDR). These analyses were done at each taxa level, whilst ANCOM was also performed at ASVs level, which confirmed the results obtained at higher taxonomic levels (data not shown).

### 2.5. Sow–Piglet Contact Had a Stronger Impact on the Nasal Microbiota of BSL3 Piglets Than the Environment

To assess the relevance of raising piglets under different environment early in life, we compared the nasal microbiota composition of BSL3-raised piglets to same-aged animals raised in farms (FB-FR). Alpha diversity estimated at a maximum even depth of 17,360 showed higher richness in L3-FC group compared to FB-FR groups, measured by Observed features and Chao1 indices (*p* < 0.01; Supplementary Figure S2A,B). L3-NC and L3-LC groups had similar richness than the whole FB-FR group, only statistically higher than one of the farms (PT, *p* < 0.05), but higher diversity when measured by Shannon’s and Simpson’s indices (Supplementary Figure S2C,D). Particularly L3-NC was statistically higher than other groups in both indices (*p* < 0.05). L3-FC diversity was statistically higher than FB-FR when computed by Shannon’s index (*p* = 0.026) that includes both richness and evenness.

**Figure 5:**
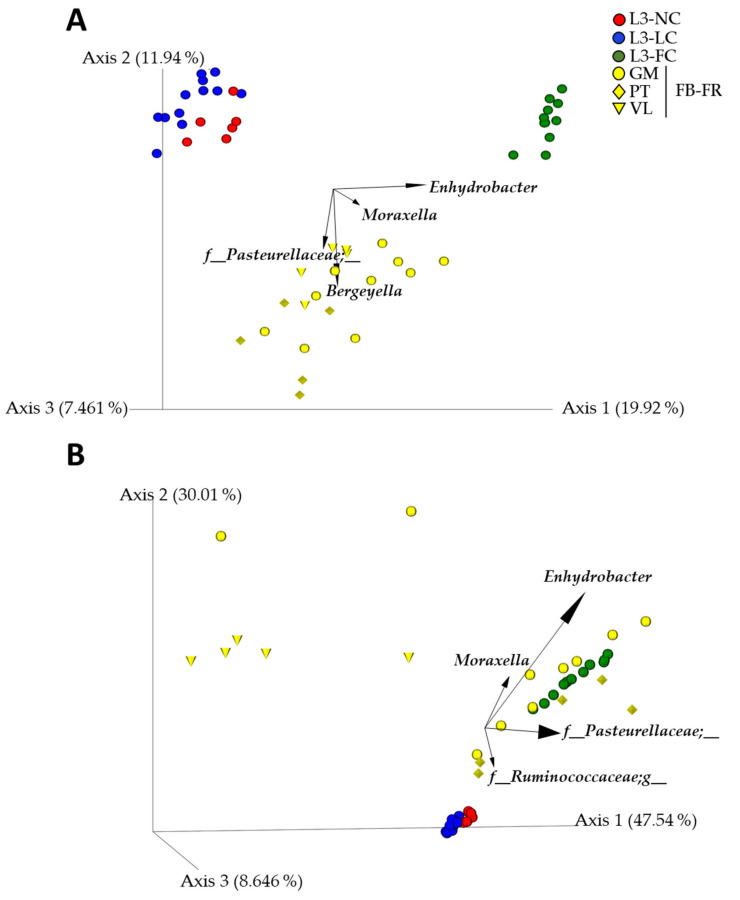
Principal component plot of the nasal microbiota of piglets raised in farms (FB-FR, in yellow) or under BSL3 conditions with different degree of contact with their sows (no contact except the birth canal (L3-NC, in red); limited contact of less than 12 h (L3-LC, in blue) and full normal contact until weaning at 3 weeks of age (L3-FC, in green)) computed through unweighted (**A**) and weighted (**B**) UniFrac analysis. The four most relevant genera explaining the differences among groups are plotted in the PCoA space. The length of each of the taxonomic vectors approximates the variance of each taxon throughout the samples. FB-FR samples have different shapes depending on the farm.

To understand compositional similarities and dissimilarities, beta diversity was estimated for FB-FR and the three BSL3 groups. All groups were statistically different in both quantitative and qualitative analyses (weighted and unweighted Unifrac, *p* < 0.01). The biplot analysis showed that *Pasteurellaceae* and *Bergeyella* shared the space with the FB-FR group, and again, *Moraxella* and *Enhydrobacter* were associated with normal sow–piglet contact (Figure 5). PCoA plots showed clustering of L3-NC and L3-LC groups when farms were included in the qualitative and quantitative analysis (Figure 5A,B), especially in the latter. This was confirmed by measuring the group distance with both metrics, where the distance among L3-NC and L3-LC groups to L3-FC was similar to the distance to FB-FR, even though the animals were all raised in BSL3.

To estimate the percentage explained by grouping animals based on the environment, they were raised and compare it to the effect of sow–piglet contact, we performed PERMANOVA tests with Adonis function with all groups. The percentage explained by grouping the animals according to the environment (BLS3 vs. FB-FR groups) was 11.29% and 17.08% in the unweighted or weighted analysis, respectively (*p* = 0.001). In addition, we evaluated the effect of different sow–piglet contact, after considering the environment (nested Adonis), where L3-FC and FB-FR were computed as a single group. The percentage of explanation by sow–piglet contact was 25.67% and 24.44% in unweighted and weighted analysis, respectively (*p* = 0.001). A normal sow–piglet contact (grouping L3-NC and L3-LC compared to L3-FC and farms) was responsible for most of the sows’ effect variation, while the mere presence of this contact (L3-NC versus the other groups) accounted for a smaller part of this effect.

**Figure 6:**
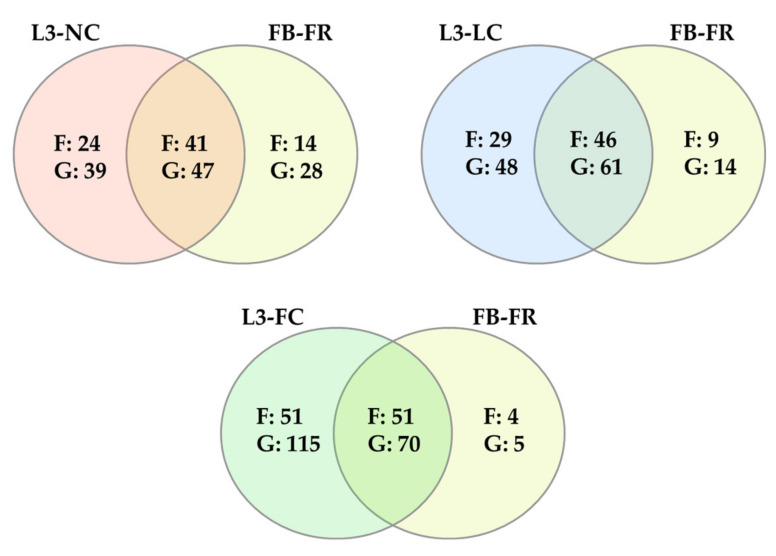
Venn diagrams of the number of taxa at genus (G) and family levels (F) found in the nasal microbiota of piglets raised in farms (FB-FR, in yellow) and under BSL3 conditions with different degree of contact with their sows. No-contact except for the birth canal (L3-NC) in red; limited contact of less than 12 h (L3-LC), in blue and full normal contact until weaning (L3-FC), in green. Only taxa present in more than 80% of samples per group were considered.

In order to qualitatively analyze the similarities among BSL3 and FB-FR groups, the core taxa of each group (represented in more than 80% of samples) were compared at family and genus levels. The number of families were 65, 75, 102 and 55; while the number of different genera were 86, 109, 185 and 75 in L3-NC, L3-LC, L3-FC and FB-FR groups, respectively. Remarkably, the longer the sow contact in the BSL3 groups, the more taxa were shared with the FB-FR group. The number of shared and exclusive taxa from each group is shown in Figure 6, while the list of common and exclusive taxa is presented in Table S3.

After confirming that L3-FC and FB-FR groups were more similar compared to other BSL3 groups (as a result of a normal contact with sows), we assessed the differential abundant taxa between these two groups, in order to explore the effect of raising piglets under BSL3 conditions. Few differences were found and most of them were detected in low abundant taxa, except for *Bergeyella*, *Rikenellaceae* and *Mycoplasma*, which were in higher relative abundance in farms, and families from *Actinobacteria*, generally more represented in L3-FC. The complete list with ANCOM results is presented in Table S4.

## 3. Discussion

The swine microbiota composition plays an important role in the physiology and immunity of the host [6,7,13,15,18,31]. Pigs are normally raised in the farms with their sows until weaning, a critical period when piglets undergo drastic changes in life conditions, and many complex diseases can arise [6,7,14,15,18,19]. Microbial colonization in early life can promote short- and long-term health benefits leading to different susceptibility to disease [18]. The sow–piglet contact and the environment have emerged as main factors influencing the microbiota composition in piglets [6,7], but in particular, the effect of growing piglets in biosecurity facilities with or without the presence of sows had not been previously assessed. Here, we found that sow–contact has more impact than the facilities environment in shaping the nasal microbiota of the piglets. The BSL3 environment increased the richness of the microbiota, probably by the colonization of transient species, which were detected in low abundance.

The nasal microbiota composition of the animals raised in BSL3 facilities was different depending on the time the piglets spent with their sows. The piglets raised with sows in the facilities showed to be dominated by *Firmicutes* and *Proteobacteria*, and in a lower relative abundance by *Bacteroidetes* and *Actinobacteria* following the tendencies observed in the healthy porcine respiratory microbiome [15]. For instance, families and genera related with swine respiratory or oral tract and sow skin microbiotas, such as *Staphylococcaceae*, *Aerococcaceae*, *Lactobacillus* or *Streptococcus* (*Firmicutes*), were more relatively abundant in piglets with normal sow contact [15,23,24,31,32]. Similarly, *Proteobacteria* was more relatively abundant in L3-FC, especially *Enhydrobacter* and *Moraxella*, both from *Moraxellaceae* family and present in the respiratory microbiota in healthy pigs [15,18,24]. In agreement, some genera such as *Moraxella*, *Rothia* and *Staphylococcus*, have been identified in teat skin and tonsils of piglets [24]. Other common members of swine microbiota, such as *Porphyromonas* and *Flavobacteriaceae* (*Bacteroidetes*) [31,32], *Corynebacterium* (*Actinobacteria*) [15,24], *Neisseriaceae* (*Proteobacteria*) [24] and *Treponema* (*Spirochaetes*) [31] or *Bergeyella* [33], *Haemophilus* [18,24] and *Mycoplasma* [22,34], were also identified as sow-derived in BSL3 samples, as they were found in the core microbiota of piglets that had contact with their sows. In contrast, an increase in *Firmicutes* and *Bacteroidetes* was found in the groups with no or limited contact with their sows (L3-NC and L3-LC), mainly due to an increase of taxa commonly found in the gastrointestinal tract of healthy pigs, such as *Lachnospiraceae*, *Ruminococcaceae* and other taxa from the *Clostridiales* order [31,35,36]; or *Prevotella* (also found in tonsils) [31,32,37], *S24-7* [38] and *Bacteroides* [31,32,36,39] from *Bacteroidales*. The richness observed in the nasal microbiota of these groups could be explained by the unusual abundance of these taxa. We hypothesize that the increased abundance of these fecal bacteria could be due to the reduced presence of sow-derived natural nasal colonizers, which would otherwise compete for the colonization, causing a decrease of community evenness, as seen in the pigs with normal contact with their sows.

The importance of sows in the development of the nasal microbiota early in life was also confirmed for BSL3 animals when the three groups were compositionally compared to normal farm samples. The normal contact with sows was more important than the environment. Importantly, we also found that the length of this contact shaped the nasal microbiota composition. Qualitative analysis showed that the longer the sow–piglet contact time was, the more common taxa were found between BSL3 and farm piglets, including swine nasal colonizers, such as *Bergeyella* [33], *Glaesserella* (*Haemophilus*) [18,24], and members from *Moraxellaceae* [15,18,24], or others found in the pig microbiota relatively abundant as *Corynebacterium* [15,24], *Treponema* [31] and *Porphyromonas* [31,32]. However, the reduced abundance of some taxa containing potential pathogens in L3-FC compared to FB-FR, such as *Pasteurellaceae* [15,18,24], *Bergeyella* [33], or *Mycoplasma* [22,34], may reflect the effect of the high biosecurity facilities as compared to the farm group. Noteworthy, *Enhydrobacter* was found in higher relative abundance in L3-FC compared to the nasal core microbiota from healthy farms [18]. Some ASVs classified as *Enhydrobacter* could really belong to *Moraxella* (as classified in the NCBI database using BLASTn; results not shown) in agreement with the current information about swine nasal microbiome [15]; however, there might also be some displacement of *Moraxella* by *Enhydrobacter* in BSL3 conditions. The inherent variability observed in farms complicates the achievement of a simple conclusion, especially in quantitative analysis where each farm is dominated by few specific taxa (possibly due to each farm’s treatment, environment, etc.). Actually, the use of antimicrobials might be also affecting the results. However, when we compared the microbial composition in animals treated with ceftiofur in both groups with altered contact (L3-LC-ceftiofur and L3-NC) and the microbiota from non-treated animals with different degree of sow–piglet contact (L3-FC and L3-LC-control), the results supported the role of the sow–piglet contact as a major driver in shaping the piglets’ microbiota.

In spite of the differences found between BSL3 groups, a common core was identified and included many taxa identified in previous studies from farm herds. Pena Cortes, et al., identified bacteria present in the vagina and teat skin of sows in strong correlation with piglet tonsillar microbiome [24] which were also identified in our nasal core microbiota of BSL3 piglets, regardless of the contact with sows. Those taxa included the *Firmicutes* families *Streptococcaceae*, *Staphylococcaceae* and *Lactobacillaceae*, as well as *Pasteurellaceae* and *Moraxellaceae* (from *Proteobacteria*), and *Micrococcaceae* (from *Actinobacteria*), and some of these were also identified in tonsils by Lowe et al. [32]. Similarly, Murase et al. [23] found Bacteroides in swine vaginal mucus or *Streptococcus*, *Moraxella* and *Rothia* in saliva, which we found in increased relative abundance in the piglets with normal contact with sows. Correa Fiz et al. also identified most of these species among the nasal respiratory core microbiota in healthy farms from Spain and the UK [18]. The vertical transmission of most of these taxa has been also described, at least, in humans [40]. Among this core, some fecal associated taxa (previously described) and other taxa that may come from food (*Cyanobacteria*) were found as well.

In this study, the nasal microbiota was assessed at the moment of weaning, which is one of the most critical moments in the development of microbial communities in piglets’ lives [6,15]. Important changes on the nasal microbiota were reported at this time-point depending on the variable contact of piglets with sows in artificially reared piglets. Nevertheless, the implications of the nasal microbiota later in life have not been assessed. Moreover, the main fact of having unbalanced microbial communities and especially the reduced contact with swine pathogens at the moment of weaning, could impair the control respiratory infections later in life [18]. Nevertheless, the health status of these animals outside this controlled environment has not been assessed. We have shown that the nasal microbiota composition changes due to the highly-controlled rearing conditions in BSL3 facilities, but more importantly, due to the reduced contact of the piglets with their mothers. The sows provide relevant colonizers to the piglets, however, some studies require to avoid sow–piglet contact and clean facilities to reproduce endemic diseases or to avoid the interaction with other farm or environmental pathogens [27,28,29]. More research is needed to unveil the implications that the distinct microbiota composition acquired in this particular environment may have on the conclusions arisen with these animal models.

## 4. Materials and Methods

### 4.1. Samples Included in the Study

The 50 samples included in the present study were taken from the nasal cavity of piglets at 3–4 weeks of age, at weaning. All piglets were normally delivered, except for L3-NC that were snatch farrowed. However, their housing and the extent of contact with their mother differed, as depicted in Table 1. Samples were from experiments previously performed with other purposes under institutional authorization and the approval of the Ethics Commission in Animal Experimentation of the Generalitat de Catalunya (Approved Protocol Numbers 9211 and 9485).

Piglets were either born and raised in farms (Farm Born-Farm Raised, FB-FR groups), born in farms but raised under BSL3 conditions (L3-NC and L3-LC), or born and raised entirely in BSL3 (L3-FC). Samples from FB-FR group came from a previous study [18] and belong to 3 farms from Catalonia (farms IDs: GM, PT and VL), which use a farrow-to-finish or multi-site production system. All farms were PRRSV stable with no clinical signs in maternity and nursery units, and had a confirmed good health status. All BSL3-raised piglets were housed in IRTA-CReSA BSL3 facilities until sampling. The time of contact with their sows (sow-contact) was an especially important variable for BSL3-raised piglets, dividing them into 3 groups. One group included piglets taken at delivery in farms (snatch farrowed), with no contact with their mothers except in the birth canal, colostrum-deprived and transferred to the BSL3 facilities for housing immediately after birth (no-contact, L3-NC). This group was entirely treated with colistin and ceftiofur to reduce mortality, since piglets were suffering from diarrhea, as they were deprived of colostrum (no maternal-derived immunity). Another group, consisted of piglets (from four different sows) that were born in farms and stayed several hours in contact with sows (less than 12h), before they were transferred to the BSL3 facilities for housing (altered-contact, L3-LC). Half of the L3-LC group was treated with ceftiofur while the other half remained untreated. The last BSL3 group consisted of piglets born from two different sows and raised in BSL3 facilities. These piglets stayed with the sows during the whole study (full-contact, L3-FC), and were untreated since it was not necessary. All sows and piglets from BSL3L groups came from the same farm. The housing in BSL3 facilities was performed according to the study groups, i.e., L3-NC piglets were housed in the same pen, L3-LC piglets were divided in two pens inside the same unit according to their treatment (ceftiofur and control), while L3-FC group was divided in two pens (one for each sow and piglets) inside the same unit. Finally, FB-FR piglets were born and grown under conventional industrial pig farming process where piglets remained with the sows for 3–4 weeks, when they were weaned.

### 4.2. DNA Extraction and 16S rRNA Gene Sequencing

Nasal samples were taken with swabs made of aluminum with a cotton tip, not flocked. The sampling procedure included the insertion of each swab into both nares. Nasal swabs were placed into sterile tube and transported under refrigeration to the laboratory, where they were resuspended (vortexed) in 500 μL of PBS and stored at −80 °C until DNA extraction. Genomic DNA was extracted with Nucleospin Blood kit (Machinery Nagel, GmbH & Co, Düren, Germany). To assess DNA quantity and quality, BioDrop DUO (BioDrop Ltd., Cambridge, UK) was used.

DNA from nasal swabs was used for 16S rRNA gene library preparation and sequencing performed at Servei de Genòmica, Universitat Autònoma de Barcelona, using Illumina pair-end 2×250 bp sequencing with MiSeq following the manufacturer instructions (MS-102-2003 MiSeq^®^ Re-agent Kit v2, 500 cycle). The target region for amplification was the variable regions V3 and V4 of the 16S rRNA gene (~460 bp), using Illumina recommended primers. The PCR product was purified and checked to verify its size on a Bioanalyzer DNA 1000 chip (Agilent). Sequences were bioinformatically sorted in samples and downstream in silico analysis was performed. The sequences analyzed in this study belong to five different runs.

All the sequences included in this study are available at the NCBI database, BioProject ID PRJNA717747.

### 4.3. Microbiota In Silico Analysis

Sequenced reads were processed using quantitative insights into microbial ecology (QIIME) 2 software version 2020.8 [41], and microbiota composition was inferred through an in-house pipeline, briefly described. First, sequenced reads were imported in QIIME2 (Qiime Import Tools) and quality was assessed with the Qiime Demux Plugin. Secondly, the DADA2 algorithm [42] was used as qiime2 plugin under the default parameters to quality filter, denoise and trim low-quality reads, remove chimeras, establishing a sequence limit length of 250bp and 240 bp for the forward and reverse reads respectively (based on quality plots), join paired-ends and sort sequences into amplicon sequence variants (ASVs). An additional quality filtering was applied using VSEARCH [43] in order to remove unspecific contaminants, where ASVs not matching latest Greengenes database (13.8) [44], provided by QIIME2 project, available at qiime2 software repository (https://docs.qiime2.org/2020.8/data-resources/, accessed on 15 February 2021); at 88% identity clustered with 65% identity and 50% query cover were filtered out with the Qiime Quality Control plugin [45]. Since the obtained reads for the samples included in this study came from five different runs, several actions were considered to deal with possible batch effects. All these steps were done individually per run in order to train the error model of this algorithm specifically based on each run’s characteristics, but ran under the same parameters so as to be comparable. After this denoising step was completed, all data was merged to proceed with the downstream analysis. Sample depths were evaluated with the Qiime Diversity Alpha-Rarefaction Plugin and normalized to an even depth in order to avoid the methodological issues [30]. Finally, after multiple aligning of sequences using MAFFT [46] and masking hypervariable positions [47] (Qiime Alignment Plugin), a phylogenetic tree was built with Fasttree [48] in order to perform further alpha and beta diversity analysis with Qiime Diversity Plugin.

Alpha diversity metrics were estimated with observed-features, Chao [49], Shannon [50], and Simpson [51] indices, and used to compute alpha group significance by pairwise non-parametric t-tests (999 random permutations) in Qiime Diversity Alpha-Group-Significance Plugin [52]. Beta diversity metrics were computed with Core-Metrics-Phylogenetic Plugin, and used to build distance matrices, in order to perform principal coordinate (PCoA) and PCoA-biplot analysis [53,54], which were visualized using Emperor [55]. Beta diversity metrics used were Unweighted UniFrac [56] ad Weighted UniFrac [57]—qualitative and quantitative, respectively. The percentage of variation in study groups was assessed with the Adonis Test Function [58] Vegan Package in R software [59]. The sample group variation was calculated by PERMANOVA pairwise test, with 999 random permutations in q2 Diversity Beta-Group-Significance Plugin [58].

Taxonomical assignment to representative sequences was obtained with the machine learning Python library Scikit-Learn using the pre-trained naïve Bayes classifier [60] trained against V3-V4 regions from 16S rRNA gene Greengenes (13.8 version) pre-clustered at 99% sequence identity, as suggested by Werner, et al. [61]. Finally, ANCOM [62] and DS-FDR [63] approaches were used to identify taxa that were differently abundant in the studied groups. The significance in the DS-FDR test was computed with a p value, while in the ANCOM test it was represented by the W score, which is a measure of how many ratio pairs of a feature (with other features) are significantly different at a fixed taxonomic level.

## 5. Conclusions

Sows are one of the most important sources of colonizers of the upper respiratory tract of piglets in early life. Artificial rearing of piglets without the presence of sows and in highly controlled environments can have a big impact on the nasal microbiota of weaning piglets and may introduce bias into research. The nasal microbial composition of piglets that had normal contact with sows is more similar to the known healthy swine nasal microbiota, while animals with altered sow–piglet contact were dominated by bacteria not commonly abundant in this body site. Understanding how sows influence the developing microbiota of piglets is a key point in understanding the swine microbiome and all the implications on piglets’ health.

## Figures and Tables

**Table 1:** Sample group distribution with main characteristics and dataset study reference.

Group	Housing	Birthplace	Sow-Contact	Perinatal Treatment	Reference
L3-NC	BSL3	Farm (snatch farrowed)	No	Colistin and crystalline ceftiofur (N = 6)	This study
L3-LC	BSL3	Farm	Less than 12 h	Crystalline ceftiofur (N = 7) Untreated (N = 6)	This study
L3-FC	BSL3	BSL3	Until weaning	Untreated (N = 11)	This study
FB-FR	Farm GM	Farm GM	Until weaning	Amoxicillin (N = 10)	[18]
	Farm PT	Farm PT	Until weaning	Not available (N = 5)	
	Farm VL	Farm VL	Until weaning	Ceftiofur and tulathromycin (N = 5)	

**Table 2:** Differently abundant taxa at phylum, order, family and genus levels found by DS-FDR Kruskal–Wallis test and ANCOM (when indicated) with the mean relative abundance for nasal microbiota of piglets raised under BSL3 conditions with different degree of contact with their sows: no contact except the birth canal (L3-NC); limited contact of less than 12 h (L3-LC) and full normal contact until weaning at 3 weeks of age (L3-FC).

Taxonomy	Relative Abundance (%)			Statistics	
Taxa *	L3-NC	L3-LC	L3-FC	DS-FDR (*p*)	ANCOM * (W)
*Acidobacteria*	1.19	0.68	0.02	0.001	
*Actinobacteria*	1.72	0.71	6.21	0.001	30
*Actinomycetales*	0.97	0.38	5.02	0.001	122
*Corynebacteriaceae*	0.03	0.04	1.03	0.001	211
*Corynebacterium*	0.03	0.04	1.03	0.001	
*Micrococcaceae*	0.34	0.24	2.92	0.001	
*Rothia*	0.20	0.24	2.76	0.001	
*Coriobacteriales*	0.26	0.05	1.11	0.001	131
*Coriobacteriaceae*	0.26	0.05	1.11	0.001	206
*Bacteroidetes*	22.53	28.78	16.54	0.001	25
*Bacteroidales*	21.46	27.84	14.62	0.001	
*Bacteroidales;f__*	1.85	4.00	2.36	0.001	
*Bacteroidales;f__;g__*	1.85	4.00	2.36	0.001	
*Bacteroidaceae*	2.11	2.85	1.29	0.001	
*Bacteroides*	2.11	2.85	1.29	0.001	
*Porphyromonadaceae*	0.81	0.95	1.70	0.001	
*Porphyromonas*	0.06	0.01	1.09	0.001	448
*Prevotellaceae*	9.28	3.46	3.46	0.002	
*Prevotella*	9.28	3.46	3.46	0.002	
*Rikenellaceae*	0.56	1.61	0.13	0.001	216
*Rikenellaceae;g__*	0.38	1.14	0.03	0.001	496
*S24-7*	3.56	5.85	1.47	0.001	
*S24-7;g__*	3.56	5.85	1.47	0.001	
*Paraprevotellaceae*	1.82	2.87	2.72	0.006	
(*Prevotella*)	1.69	2.34	1.46	0.001	
*p-2534-18B5*	0.48	5.53	0.10	0.001	248
*p-2534-18B5;g__*	0.48	5.53	0.10	0.001	505
*Flavobacteriales*	0.43	0.76	1.76	0.002	
*Flavobacteriaceae*	0.16	0.19	1.19	0.001	
*Cyanobacteria*	2.77	3.71	0.59	0.001	29
*Firmicutes*	58.43	50.69	37.69	0.027	
*Bacillales*	0.36	0.56	1.53	0.001	
*Staphylococcaceae*	0.26	0.55	1.12	0.001	
*Lactobacillales*	1.23	1.68	5.00	0.001	
*Aerococcaceae*	0.06	0.01	1.31	0.001	224
*Lactobacillaceae*	0.20	0.76	1.01	0.001	
*Lactobacillus*	0.20	0.76	1.00	0.001	
*Streptococcaceae*	0.73	0.49	1.62	0.001	
*Streptococcus*	0.59	0.42	1.52	0.001	
*Clostridiales*	54.65	45.83	28.82	0.001	
*Clostridiales;f__*	7.58	8.77	2.46	0.001	
*Clostridiales;f__;g__*	7.58	8.77	2.46	0.001	
*Christensenellaceae*	1.51	0.26	0.92	0.001	
*Christensenellaceae;g__*	1.51	0.26	0.92	0.001	
*Clostridiaceae*	1.14	0.67	1.53	0.001	
*Lachnospiraceae*	17.25	11.67	5.94	0.001	
*Lachnospiraceae;__*	3.77	2.79	1.23	0.001	
*Lachnospiraceae;g__*	5.76	4.68	2.07	0.001	
*Blautia*	1.23	0.41	0.50	0.001	
*Coprococcus*	2.93	1.48	0.91	0.001	
*Roseburia*	1.22	0.17	0.23	0.001	
*Ruminococcaceae*	21.77	19.88	10.86	0.001	
*Ruminococcaceae;__*	1.61	1.71	0.75	0.001	
*Oscillospira*	3.87	6.03	1.24	0.001	
*Ruminococcus*	4.41	3.52	1.27	0.001	
(*Mogibacteriaceae*)	0.89	0.52	1.20	0.001	
(*Mogibacteriaceae*);g__	0.87	0.52	1.19	0.001	
(*Tissierellaceae*)	0.14	0.05	1.15	0.007	
*Proteobacteria*	8.21	9.46	31.06	0.001	27
*Rhizobiales*	0.95	1.18	0.16	0.001	123
*Neisseriales*	0.05	0.37	1.03	0.001	143
*Neisseriaceae*	0.05	0.37	1.03	0.005	241
*Pseudomonadales*	1.38	2.19	26.17	0.001	135
*Moraxellaceae*	1.15	2.01	26.01	0.002	216
*Moraxellaceae;__*	0.12	0.37	1.41	0.001	
*Enhydrobacter*	0.34	0.23	18.32	0.001	450
*Moraxella*	0.44	0.77	5.98	0.001	
*Spirochaetes*	0.38	0.77	2.56	0.001	29
*Spirochaetales*	0.09	0.64	2.01	0.001	145
*Spirochaetaceae*	0.09	0.64	2.01	0.001	249
*Treponema*	0.09	0.64	2.01	0.001	493
*Tenericutes*	1.38	1.66	0.66	0.001	27
*Verrucomicrobia*	0.68	0.59	1.55	0.001	
*WCHB1-41*	0.00	0.16	1.18	0.001	144
*RFP12*	0.00	0.15	1.08	0.001	249
*RFP12;g__*	0.00	0.15	1.08	0.001	522

* The brackets indicate that the taxonomic name is contested.

## Data Availability

All the sequences included in this study are available at the NCBI database, BioProject ID PRJNA717747.

## Supplementary Materials

The following are available online at https://www.mdpi.com/article/10.3390/pathogens10060697/s1, Figure S1: Taxonomical composition at (A) phylum and (B) family levels of the nasal microbiota of piglets raised in BSL3 conditions with variable contact with sows: L3-NC, no contact except the birth canal; L3-LC, limited contact of less than 12 h; and L3-FC, full normal contact until weaning at 3 weeks of age. Only taxa > 1% global relative abundance in at least one group is represented. *Families* belonging most relatively abundant *Phyla* have been colored in common color-scheme to simplify its visualization; green for *Firmicutes*, blue for *Bacteroidetes*, and red for *Proteobacteria*. The legend shows the taxa ordered by global relative abundance from bottom to top. Figure S2: Alpha diversity of the nasal microbiota of piglets raised under BSL3 conditions with different degree of contact with their sows (no contact except the birth canal (L3-NC, in red); limited contact of less than 12 h (L3-LC, in blue) and full normal contact until weaning at 3 weeks of age (L3-FC, in green)); and piglets from conventional farm industry (FB-FR, in yellow). Group FB-FR included piglets from three farms (GM, PT and VL). Alpha diversity was measured at a depth of 17,360 by observed ASVs (A), Chao1 (B), Shannon–Weaver (C) and Simpson (D) indices. Significant Kruskal–Wallis pairwise tests are depicted in the top bars. Table S1: Taxonomical composition at phylum, family and genus levels of the nasal microbiota from piglets raised in BSL3 conditions with variable contact with sows. L3-NC, no contact with the sows except the birth canal; L3-LC, limited contact of less than 12 h; and L3-FC, full normal contact until weaning at 3 weeks of age. Only taxa with global relative abundance higher than 1% in at least one group is included. All taxa with less relative abundance than 1% have been grouped. Table S2: Distribution of the taxa at family and genus levels found in piglets raised under BSL3 conditions with different degree of contact with their sows, and depicted in Figure 4: no contact except the birth canal (L3-NC); limited contact of less than 12 h (L3-LC) and full normal contact until weaning at 3 weeks of age (L3-FC). Only taxa present in more than 80% of samples in a group were included in the analysis. Table S3: Taxa at family and genus levels found in the nasal microbiota of piglets raised in farms (FB-FR) or under BSL3 conditions with different degree of contact with their sows, depicted in Figure 6. No-contact except for the birth canal (L3-NC); limited contact of less than 12 h (L3-LC), and full normal contact until weaning (L3-FC). Only taxa present in more than 80% of samples per group were considered. Table S4: Differently abundant taxa at phylum, family and genus levels found by ANCOM with the mean relative abundance for nasal microbiota of piglets raised in farms (FB-FR) or under BSL3 conditions with full normal contact until weaning at 3 weeks of age (L3-FC). Relative abundance of each taxon from individual farms (GM, PT and VL) is also included.
