# Supplementary figures and images for: Sow Contact Is a Major Driver in the Development of the Nasal Microbiota of Piglets
Source: Pathogens. 2021 Jun 3;10(6):697. doi: 10.3390/pathogens10060697 (PMC8227386; doi:10.3390/pathogens10060697)

A

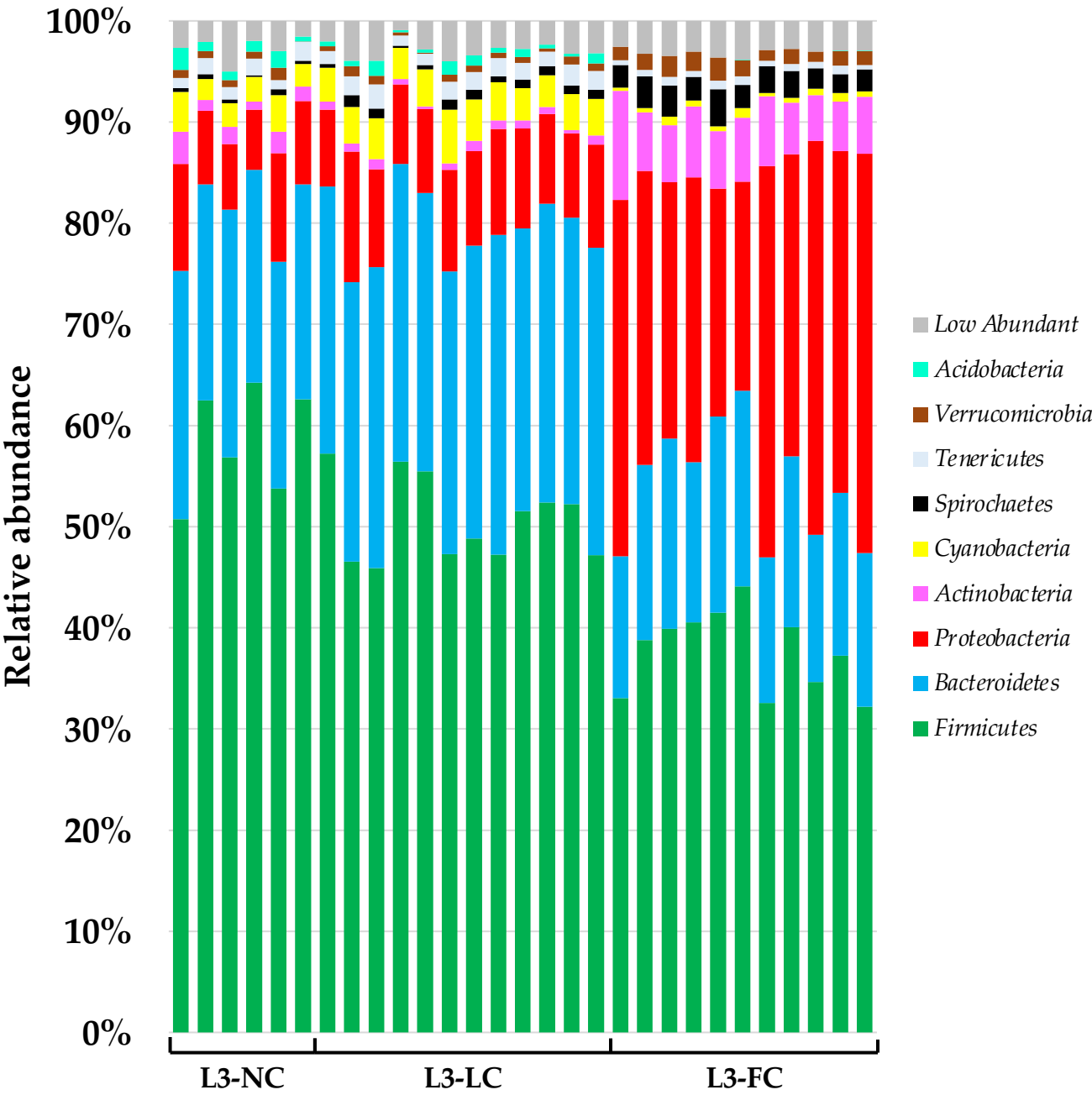

Supplement: Supplementary file 1 [file pathogens-10-00697-s001.zip › supplemetary_material/figureS1A.pdf]

**B**

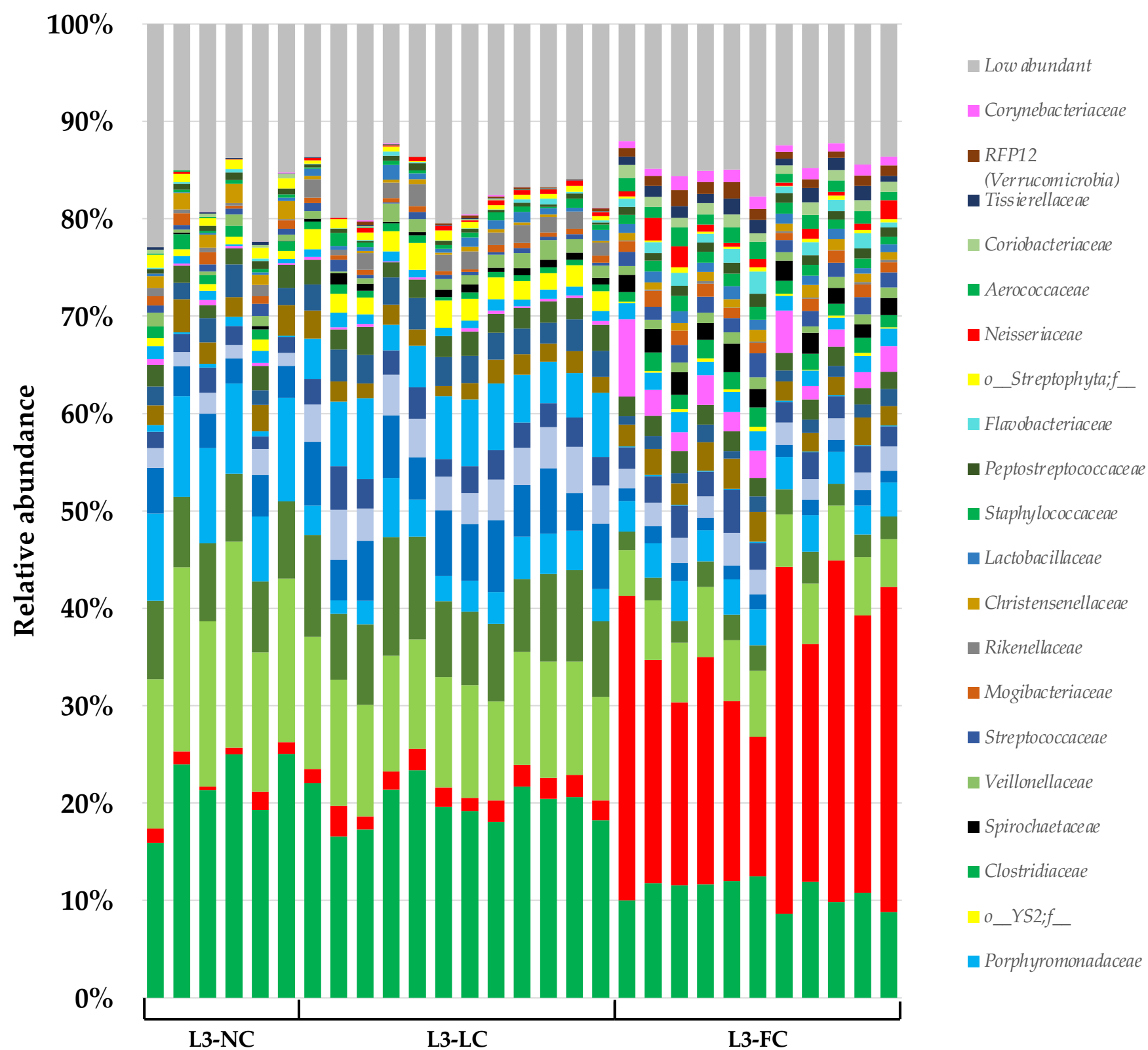

Supplement: Supplementary file 1 [file pathogens-10-00697-s001.zip › supplemetary_material/figureS1B.pdf]

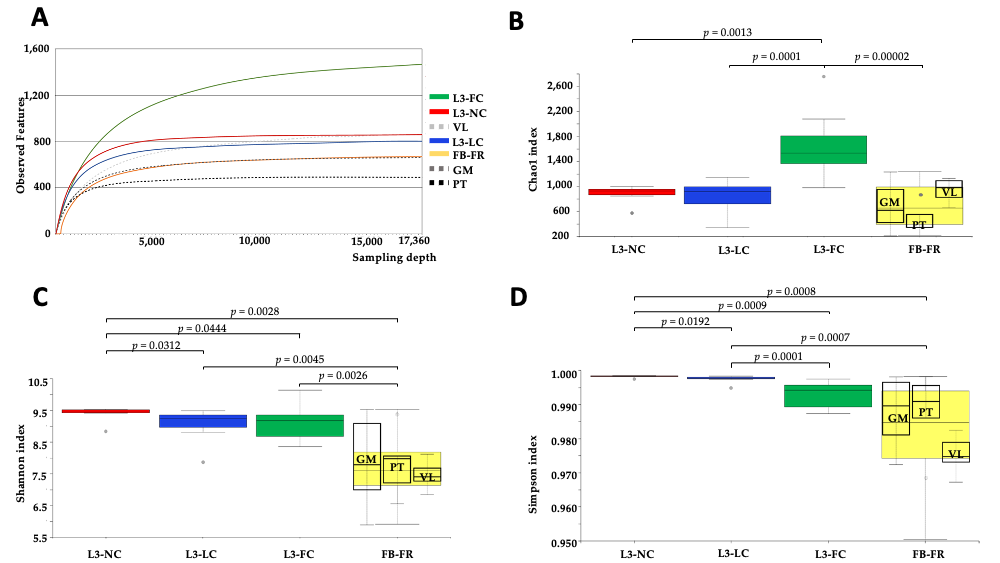

Supplement: Supplementary file 1 [file pathogens-10-00697-s001.zip › supplemetary_material/FigureS2.tiff]
